# Supplementary figures and images for: A combination strategy for enhancing linkage to and retention in HIV care among adults newly diagnosed with HIV in Mozambique: study protocol for a site-randomized implementation science study
Source: BMC Infect Dis. 2014 Oct 15;14:549. doi: 10.1186/s12879-014-0549-5 (PMC4210581; doi:10.1186/s12879-014-0549-5)

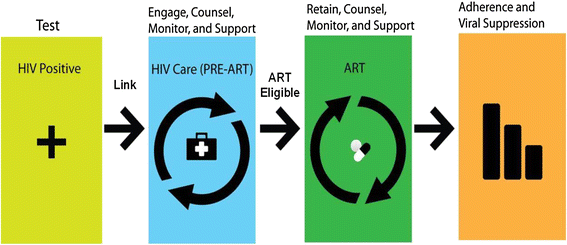

Supplement: Supplementary file 1 — Authors’ original file for figure 1 [file 12879_2014_549_MOESM1_ESM.gif]

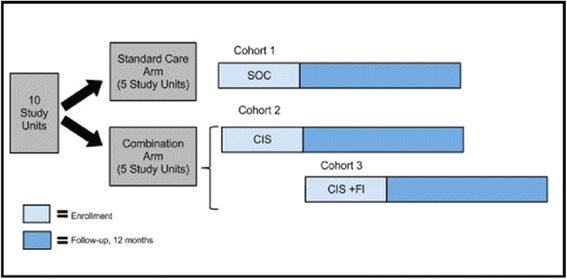

Supplement: Supplementary file 2 — Authors’ original file for figure 2 [file 12879_2014_549_MOESM2_ESM.gif]
